# Management of Loyalty and Its Main Antecedents in Sport Organizations: A Systematic Analysis Review

**DOI:** 10.3389/fpsyg.2021.783781

**Published:** 2021-11-25

**Authors:** Cristina Loranca-Valle, Pedro Cuesta-Valiño, Estela Núnez-Barriopedro, Pablo Gutiérrez-Rodríguez

**Affiliations:** ^1^Department of Economy and Business Management, Universidad de Alcalá, Alcalá de Henares, Spain; ^2^Department of Business and Economy Management, Universidad de León, León, Spain

**Keywords:** loyalty, sport management, satisfaction, service quality, commitment, trust, systematic review

## Abstract

Sports management is booming, thanks to society’s growing interest in sports in general. This work provides an exhaustive review of the scientific literature of one of the variables most coveted by managers: loyalty. Antecedents positively related to loyalty are also conceptualized in this paper. The scientific literature search was limited to the last 15years to achieve a better fit with today’s context. The Web of Science database was the main search method used, along with other, secondary resources, for a total of 328 scientific articles obtained as a base for this review. The results of this study bring together the most representative data in a systematic review of the loyalty variable in the field of sports management: the antecedents of loyalty, the terms with the greatest presence in the theory, the most representative authors and sources of this topic, and so on. One of the most significant results obtained from the review of these 300 bibliographical references is that the factors to be borne in mind for marketing strategies in sports organizations are satisfaction, commitment, trust, and service quality. The literature reflects that these variables, present in studies on loyalty in general, can also be found in the literature on sports management – in particular, satisfaction and service quality. However, few papers include all these antecedents at once, making this an interesting field for future research.

## Introduction

Loyalty is one of the principal objectives of marketing and is sometimes even equated to the concept of marketing itself (Sheth, 1996). Loyalty is a variable that has been extensively researched in the literature of psychology, marketing, and management, in which many authors have studied the variables that explain loyalty (Dwyer et al., 1987; Dick and Basu, 1994; Sheth, 1996; Núñez-Barriopedro et al., 2021). There are, however, three variables that are repeated in many proposals and models: satisfaction, trust, and commitment (Garbarino and Johnson, 1999; Hennig-Thurau et al., 2001; Bansal et al., 2004; Rauyruen and Miller, 2007). Service quality is an another variable that most commonly appears in the literature on consumer loyalty, and it is included by authors such as (Cronin and Taylor, 1992; Hur et al., 2011; Kim and Trail, 2011; Mandhachitara and Poolthong, 2011; Pérez-Cabañero et al., 2017). Most authors who study loyalty from a marketing viewpoint do so, firstly, from the perspective of behavioral loyalty, i.e., repeating the exchange relationship (Chaudhuri and Holbrook, 2001; Szymanski and Henard, 2001; Chiou and Drogue, 2006), and secondly, from the perspective of attitudinal loyalty (Kunkel et al., 2013, 2019).

The sports industry generates billions of dollars; a better understanding of the relationship between the product and its consumers is thus a key factor for the marketing managers of sport organizations. Maintaining relationships over time, independently of whatever factors may arise, is also of key importance. Loyalty therefore becomes a crucial element in maintaining relationships over time and thus an element on which marketing managers must focus (Tsiotsou, 2013).

Sport organizations include all those bodies that pursue sports-related activities. They may therefore be companies that offer goods (sports clothes, sports apparatus and materials, etc.), services (personal training, broadcasting games on television, etc.), ideas (consequences of belonging to a club or taking part in a sport: slimming, giving members an identity), or a combination of all of these (Smith and Stewart, 2013). The way loyalty is explained may vary as a function of the type of sports product in question and also of the different types of consumer – such as those attending events, those watching, listening to, or reading the media, those purchasing products with licenses, etc. (Kim et al., 2011).

Sports products have unique aspects that affect the basic conditions for selling them: they are products that are inherently uncertain; they are inconsistent and open to subjective interpretations; they take place under highly competitive conditions; and marketing managers for these products do not have full control over the marketing mix (Mullin et al., 2007). It is also true, however, that it has been shown empirically that members of sport organizations tend to behave in their sport and its money-making activities in the same way as in other areas of consumption (Bodet, 2012).

Consumer loyalty is an asset highly valued by all types of organizations and directing available resources toward improving loyalty is much easier if this concept has been analyzed. This work aims to examine the existing literature on loyalty to a sports organization and verify whether the variables that explain loyalty in organizations in general are also present in sports theory. On this basis, the following questions are raised:

Q1: Does loyalty to sports organizations behave in the same way as consumer loyalty to any other organizations?

Q2: Is the quality of sports services a key variable for consumer loyalty?

Q3: Is satisfaction the most representative variable of sports consumer loyalty?

Q4: Can commitment and trust variables explain loyalty in sports?

The following sections aim to offer an exhaustive review of loyalty from the point of view of sports management considering the main antecedents that lead to loyalty in sports organizations. In a first step, the different loyalty concepts and their main antecedents (satisfaction, service quality, commitment, and trust) are analyzed. Then the various sources used are detailed, as well as the main search criteria. The results presented compile the most notable data on the bibliography related to sports loyalty, through a systematic analysis of the literature, while the paper ends with the main conclusions derived from this search.

## Concept of Loyalty and its Main Antecedents

Loyalty has been conceptualized by various authors in the fields of both psychology and marketing. Many authors have made in-depth studies of this concept from the marketing perspective, and all agree that loyalty comprises all those behaviors involving same-brand purchasing repeatedly, both now and in the future (Dick and Basu, 1994; Oliver, 1999; Chaudhuri and Holbrook, 2001; Hennig-Thurau et al., 2002; Cuesta-Valiño et al., 2021a), despite situational influences and marketing efforts that have the potential to cause switching behavior (Oliver, 1999).

Loyalty is made up of two concepts, which combine to give the variable the greatest explanatory power. These two concepts are: behavioral aspects or purchase intentions and attitudinal loyalty (Rauyruen and Miller, 2007; Cossío-Silva et al., 2016). Behavioral loyalty has been identified as customers’ willingness to repurchase the product or the services and to maintain a relationship with the supplier or service provider. Attitudinal loyalty, however, is the consumer’s level of commitment toward a product/brand and their promotional attitude toward the supplier or service provider (Dick and Basu, 1994; Chaudhuri and Holbrook, 2001).

By focusing on loyalty in the field studied in this paper, i.e., sport, it becomes clear that it is more difficult to conceptualize loyalty when the subject is services than when discussing products. This is partly because, in the case of services, it is impossible to separate the consumer from the person providing the service, and this participation by the client in the interpersonal component of services – their production and delivery – adds emotional dimensions to loyalty (Lewis and Soureli, 2006). The individual’s underlying willingness to repurchase, together with evaluation of the alternatives, is inseparable from the notion of loyalty (Jacoby and Kyner, 1973).

In recent decades, the literature on loyalty has multiplied and we have found many studies that aim to explain it (see Table 1). These ideas are summarized in the sections below, which explain the principal antecedents of this variable.

**Table 1:** Loyalty definitions.

References	Definition
Chaudhuri and Holbrook (2001)	Behavioral loyalty is “the willingness of average business customers to repurchase the service and the product of the service provider and to maintain a relationship with the service provider/supplier.” Attitudinal loyalty is “the level of customer’s psychological attachments and attitudinal advocacy towards the service provider/supplier.”
Rauyruen and Miller (2007)	
Oliver (1999)	“To re-buy or re-patronize a preferred product/service consistently in the future, thereby causing repetitive same-brand or same brand-set purchasing, despite situational influences and marketing efforts having the potential to cause switching behavior”
Pressgrove and Mckeever (2016)	“Loyalty is a complex multidimensional variable with little consensus concerning the specific dimensions and how they interact to determine a behavioral outcome”

### Service Quality

This variable is defined as the discrepancy between a consumer’s prior expectations for a given service and his or her perception regarding the purchase or services (Parasuraman et al., 1988; Boulding et al., 1993; Hennig-Thurau et al., 2002). This implies that, for the same perception, the higher the consumer’s expectations the lower the perceived quality will be (Parasuraman et al., 1988).

Parasuraman et al. (1985) created a scale to measure quality based on 10 dimensions, which were subsequently summarized into five: (1) tangibles, (2) reliability, (3) responsiveness, (4) assurance, and (5) empathy (Parasuraman et al., 1988). This scale has been the basis of numerous studies on quality in different settings (Gilbert and Wong, 2003; Lee-Ross, 2008; Butt and de Run, 2010; Samen et al., 2013; Farooq et al., 2019), although it has also been criticized by many authors (Cronin and Taylor, 1992; Buttle, 1996; Robledo, 2001). Several authors have created models to explain service quality in specific sectors (Chang and Yeh, 2002; Chen and Chen, 2010; Meesala and Paul, 2018; Hult et al., 2019; Cuesta-Valiño et al., 2020; see Table 2).

**Table 2:** Perceived Quality definitions.

References	Definition
Boulding et al. (1993)	“The consumer’s perception of overall service quality results from a comparison between expectations and perceptions of the different components of service. With perceptions of services held fixed, the higher the expectations, the lower the perceived quality”
Parasuraman et al. (1988) Su et al. (2016)	“It is a form of attitude, related but not equivalent to satisfaction, and results from a comparison of expectations with perceptions of performance”
Zeithaml (1987)	“Perceived Quality is the consumer’s judgment about an entity’s overall excellence or superiority”

### Satisfaction

General satisfaction is a general assessment based on all the purchases and the consumers’ experience of using a product or service (Fornell, 1992; Anderson et al., 1994). Another definition of satisfaction is customer’s emotional or sentimental reaction to the perceived differences between expectations and actual implementation (Oliver, 1980; Andreassen, 2000). This definition may cause confusion between satisfaction and perceived quality, but these variables are different. The principal difference is that satisfaction is a type of attitude, a perception over the long term, while perceived quality measures a specific transaction (Parasuraman et al., 1988). Zablah et al. (2016) define satisfaction as “subjective evaluation made as a post-choice cognitive judgment,” a meaning that follows the same emotional line of Trail et al. (2000) and Maxham and Netemeyer (2002); see Table 3).

**Table 3:** Definitions of satisfaction.

References	Definition
Andreassen (2000)	“Satisfaction is consequently related to providing what is being sought to the point where fulfillment is reached”
Oliver (1981)	“Summary psychological state resulting when the emotion surrounding disconfirmed expectations is coupled with the consumer’s prior feelings about the consumption experience”
Sanz-Blas et al. (2014)	“Satisfaction is defined as the contentment of the customer with respect to his or her prior g experience”

### Commitment

Commitment is understood to be the implicit or explicit promise of a long-term relationship between the parties (Geyskens et al., 1996; Ruyter et al., 1998; Dwyer et al., 2015). Simplifying the equation, one can consider commitment to be the motivation that pushes a party to trust a given company (Moorman et al., 1993) or the psychological attachment to the organization (Gruen et al., 2000). Commitment implies the conviction by both parties that maintaining the relationship will be more beneficial than ending it (Geyskens et al., 1996). All definitions of commitment agree that there is a psychological component (bond, link, promise, or dedication) and a motivational component (maintaining the relationship; repeated purchasing; remaining in the organization; Jones et al., 2010). Commitment and sport are closely related: indeed, there are multiple works that study the relationship of commitment between the athlete and the sports organization (Mahony et al., 2000; Ross et al., 2006; Rather, 2018; Cuesta-Valiño et al., 2021b). The different meaning of commitment are compiled in Table 4.

**Table 4:** Commitment definitions.

References	Definition
Gruen et al. (2000)	“The degree of the membership’s psychological attachment to the association”
Moorman et al. (1993)	“An enduring desire to maintain a valued relationship”
Morgan and Hunt (1994)	“An exchange partner believing that an ongoing relationship with another is so important as to warrant maximum efforts at maintaining it”

### Trust

Many authors agree that trust is one of the basic ingredients for success in relationships (Dwyer et al., 1987; Moorman et al., 1993; Berry, 1995). Trust in an organization is based on the consumer’s certainty of the quality and integrity of the service offered (Moorman et al., 1993; Morgan and Hunt, 1994; Garbarino and Johnson, 1999; Hennig-Thurau et al., 2001). Trust is the belief by one of the parties that the actions the other party takes will necessarily satisfy his/her needs (Anderson and Weitz, 1989). In relationships between the consumer and companies, the psychological benefits of security and trust are more important than special treatment or the social benefits arising from that relationship (Gwinner et al., 1998). Trust is an involvement in a process, which has been well thought-out and carefully considered, while brand attachment or affect is spontaneous, more immediate, and less reasoned (Chaudhuri and Holbrook, 2001). Trust, like commitment may have both affective and cognitive dimensions (Johnson and Grayson, 2005). Trust is a positive attribute that gives the members of an organization the necessary commitment to carry out the objectives set (Józefowicz, 2013; see Table 5).

**Table 5:** Trust definitions.

References	Definition
Chaudhuri and Holbrook (2001)	“The willingness of the average consumer (user/donor) to rely on the ability of the brand (company/organization) to perform its stated function”
Morgan and Hunt (1994)	“A key condition in the long-term development of relationships. High levels of trust are said to reduce uncertainty and diminish perceptions of risk in a relationship”
Sirdeshmukh et al. (2002)	“Trust as having two components: performance or credibility trust and benevolence trust”

## Materials and Methods

Following other examples (Montero and López-Sánchez, 2021; Södergren, 2021), a systematic review of the scientific literature was carried out to identify, classify, and synthesize the existing publications on the loyalty variable in a sports context. The procedure had three steps: identify the literature, extract the data, and present results. In the search stage, the possible articles most suitable for this study were identified using different data sources, such as Google Scholar, Scopus Sage Journals and – above all –Web of Science (WoS). WoS was used as the main source because it contains the publications with the highest impact index, as well as being one of the most extensive databases. Its advanced search formula allows large specifications and helps to classify the studies analyzed.

**Figure 1:**
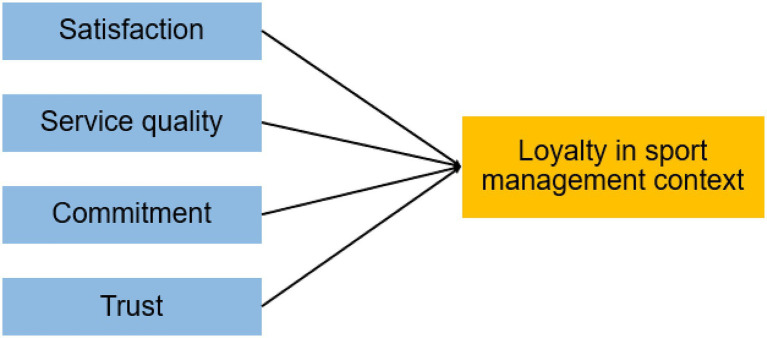
Loyalty and its antecedents in a sport management context.

Loyalty has been a highly studied concept in different areas, that is why the search criteria used have been very specific. Using key words like “loyalty and sport,” “loyalty and sport management,” “loyalty and satisfaction in sport management,” “loyalty and service quality in sport management,” “loyalty and commitment in sport management,” and “loyalty and trust in sport management.” Satisfaction, service quality, commitment, and trust were included in the search criterion because they are the main variables that affect to loyalty in a general context. Figure 1 represents the idea of loyalty and its antecedents transferred to a sport management context.

The search time range covered the last 15years, from 2006 to 2021. During that period, sport has experienced significant growth – as have all related sectors – and competition between organizations is getting increasingly strong, leading in turn to an increased need to retain the consumer (Wemmer and Koenigstorfer, 2016; Cuesta-Valiño et al., 2021c).

Some 1,154 articles were compiled in the identification phase. These were subsequently reduced to 599 once duplicate articles were eliminated. Several refining phases followed, with the analysis of the abstracts and the keywords included in each article.

**Figure 2:**
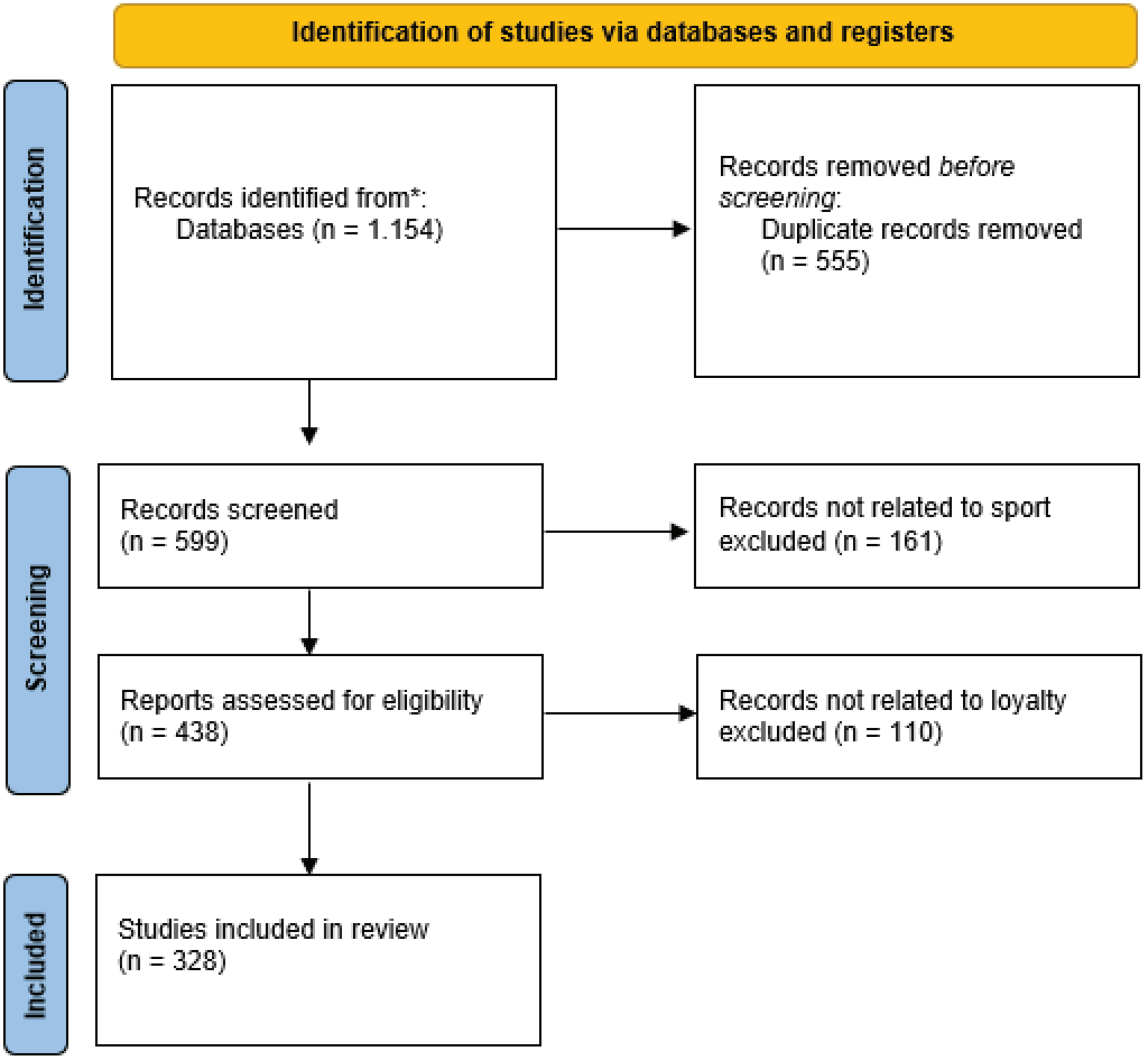
PRISMA 2020 flow diagram for new systematic reviews.

In a first step, all those articles not specific to the sports sector were excluded, leaving the database with 438 articles. In a second exclusion phase, all those articles that did not measure the loyalty variable were rejected: 328 articles remained. Figure 2 specifies the flow chart followed in the selection process.

The selection, classification, and exclusion phases were carried out using an Excel file. In the following section, the data extracted – mainly from the WoS – are presented.

## Result of Bibliometric Analysis

The results of this review indicate that there are various authors who have vast expertise in sports loyalty. Table 6 shows the authors with the highest number of published articles and the most cited authors among the articles analyzed.

**Table 6:** Number of papers and citations per author.

Author	Number of publications
Funk, Daniel C.	15
Doyle, Jason	10
McDonald, Heath	9
Kunkel, Thilo	9
García-Fernández, Jerónimo	8
**Author**	**Number of citations**
Funk, Daniel C.	473
Yoshida, Masayuki	274
Heere, Bob	259
McDonald, Heath	227
Gordon, Brian	205

There are numerous magazines that focus on sports and sports management. Moreover, articles on sports loyalty are also published in other journals, such as those with a more general focus or in other specific fields such as tourism or the internet. The magazines that publish the most articles on the subject studied are mainly magazines specific to sports management. Table 7 shows the five journals that publish the most articles on this topic. However, the most cited magazines regarding loyalty in sport have other approaches.

**Table 7:** Number of publications and citations per journal.

Journal	Number of publications
Sport Management Review	34
Journal of Sport Management	19
International Journal of Sports Marketing & Sponsorship	10
European Sport Management Quarterly	9
Sport Business and Management- An International Journal	9
**Journal**	**Number of citations**
Sport Management Review	1,034
Journal of Sport Management	929
Internet Research	192
Journal of Sustainable Tourism	180
Journal of Travel Research	156

**Figure 3:**
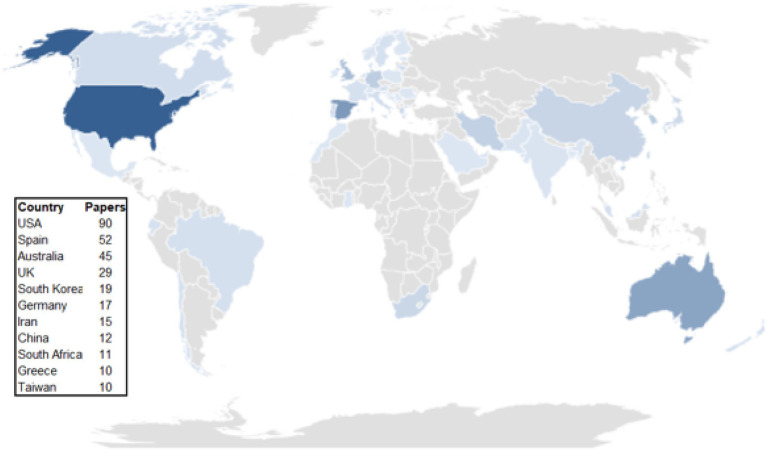
Geographic distribution of loyalty and sport management.

Figure 3 shows the countries that publish contents on loyalty in sport. It is a color map and the stronger color used for a certain country, the more papers published in that country. As can be seen, the United States is the country that has produced the most papers (90 articles), followed by Spain (52), Australia (45), United Kingdom (29) and South Korea (19).

In the literature there are different terms related to loyalty that appear in some of the most accepted definitions of loyalty in the theory, although such definitions differ. An example is the term “purchase intentions” defined in loyalty as the intention to buy from the same organization continuously over time (Oliver, 1999; Chaudhuri and Holbrook, 2001; Rauyruen and Miller, 2007).

Another term that can be linked to loyalty is “resistance to change,” since this concept appears in various definitions of loyalty (Oliver, 1999).

Resistance to change was a more common term in the literature than loyalty; however, in recent years loyalty has become much more popular, surpassing resistance to change.

**Figure 4:**
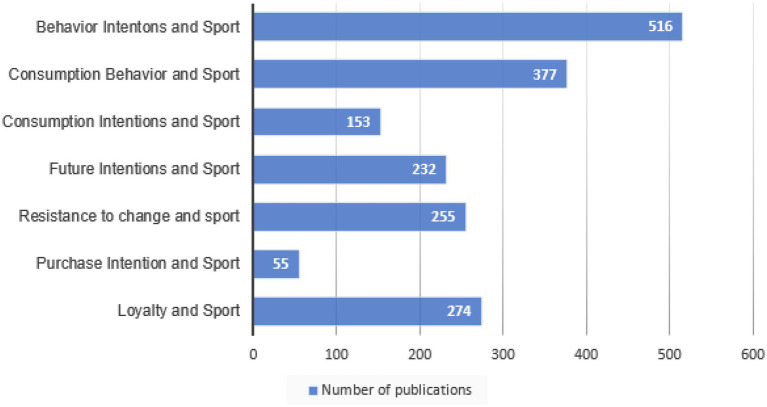
Number of papers for loyalty in sport management and other terms related to loyalty.

Without any doubt, the term most used in theory is “behavior intentions,” followed closely by “consumption behavior.” Nevertheless, these two terms are much more general than loyalty and resistance to change, since they include loyalty and other variables such as word of mouth. (Boulding et al., 1993). In addition, most of the records obtained with the keyword “behavior intentions” and “consumption behavior” are duplicated in the other terms analyzed. Figure 4 presents all mentioned terms.

It is undoubtedly true that all types of sports organizations aspire to gain the loyalty of sports consumers. The literature includes various examples of the variables that explain this loyalty. The most common loyalty antecedents in the general literature are satisfaction, quality of service, commitment, and trust. The question is whether this also occurs in the literature on loyalty in sport.

**Figure 5:**
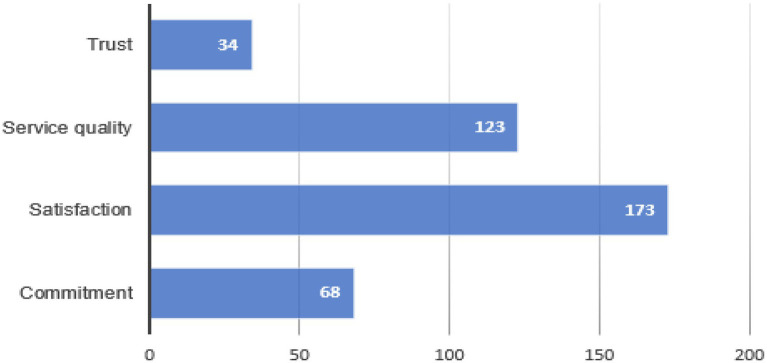
Distribution of antecedents that explain loyalty over total publications.

Satisfaction is the variable most present in the literature on loyalty in the sports field. Another of the variables that has been most associated with loyalty is service quality. Figure 5 shows the percentage represented by each of the variables that explain loyalty in the articles studied.

Although the variables most found in the literature on loyalty in sport are satisfaction and service quality, the articles with the most citations do not study these variables, focusing instead on commitment and trust.

In total, there are 328 articles on loyalty in sport, of which five are those that study the four variables analyzed in this paper: satisfaction, service quality, commitment, and trust. Table 8 shows the five papers that includes all these variables.

**Table 8:** Articles that include all analyzed variable.

Title	References	Citations
Sustainable Management of Sports Federations: The Indirect Effects of Perceived Service on Member’s Loyalty	Cuesta-Valiño et al. (2021c)	0
Built to last: relationship quality management for season ticket holders	Lee et al. (2020)	7
Influence of Brand Image of a Sports Event on the Recommendation of Its Participants	Martínez Cevallos et al. (2020)	2
Perceived value, satisfaction and future intentions in sport services Putting congruence and brand trust in the equation - linear models vs. QCA	Alguacil et al. (2019)	2
The Impact of Perceived Service Quality on Customer Loyalty in Sports Clubs	Schijns et al. (2016)	4

## Discussion and Conclusion

### Theorical and Managerial Discussions

The loyalty variable has always been extensively researched in the field of literature of business field and more specifically in the sports field. Indeed, there is so much literature associated with loyalty in the field of sports management that it can be very difficult to navigate. The purpose of this paper, then, is to use a systematic review of more than 300 references to identify and classify the most relevant information on this topic and review the different factors and variables that define loyalty in sports management. Finally, it aims to check if these variables coincide with the most common variables in loyalty in general. This in turn requires an in-depth study of the variables that influence the loyalty.

Satisfaction is a crucial element of consumer loyalty; most studies that model loyalty include satisfaction as an explanatory variable, and, in practically, all cases they demonstrate its positive influence (Dick and Basu, 1994; Oliver, 1999; Eriksson and Löfmarck Vaghult, 2000; Geyskens and Steenkamp, 2000; Rauyruen and Miller, 2007; Foroudi et al., 2016; Henseler et al., 2016; Cuesta-Valiño et al., 2019; Gutiérrez Rodríguez et al., 2020). Despite this, many authors consider that the relationship between satisfaction and loyalty is more complicated than it appears to be (Reichheld, 1993; Stauss and Neuhaus, 1997; Oliver, 1999; Bodet, 2012); satisfaction has a significant emotional component (Rauyruen and Miller, 2007) and this is influenced by the other variables (Parasuraman et al., 1988; Shane and Ulrich, 2004; Chiou and Drogue, 2006; Nemati et al., 2010; López-Sanz et al., 2021). In many cases, it acts as a mediating variable for other variables (Chiou and Drogue, 2006; Demirci and Kara, 2014; Abrudan et al., 2015; Foroudi et al., 2016; Kashif et al., 2016; Su et al., 2016), usually service quality. Service quality is clearly connected to satisfaction (Parasuraman et al., 1988; Pugh, 2001; Loranca-Valle et al., 2019) and both directly and indirectly to loyalty (Cronin and Taylor, 1992; Mandhachitara and Poolthong, 2011). These two variables are very present in the literature on sports management; in fact, taken together they explain loyalty in almost 75% of publications in this area. We find many examples of jobs both in the private sector (Bodet, 2012; Haro-González et al., 2018; Castillo-Rodríguez and Onetti-onetti, 2019) and in the public sector (García-Fernández et al., 2018; Cuesta-Valiño et al. 2021c).

The variables commitment and trust are much less present in the literature on loyalty in sports management than in other sectors such as the workplace. However, they cannot be ignored since articles that include these two variables in relation to loyalty have the best citation ratios: a very good indicator of the quality of these works. These two variables are definitely related to each other (Williamson, 1985; Gundlach et al., 1995; Garbarino and Johnson, 1999) and are also related to loyalty, especially in the case of non-profit organizations (Gruen et al., 2000; Galan-Ladero et al., 2013; Pressgrove and Mckeever, 2016; Barra et al., 2018) and those that are also sports-related (Ferrand et al., 2010; Kim et al., 2011). The two dimensions of both variables – cognitive and affective – are taken into account (Kumar et al., 1994; Johnson and Grayson, 2005; Rauyruen and Miller, 2007; Jones et al., 2010).

The country that produces the most publications on loyalty and sports management is the United States, which has 92 articles. This can logically be expected, since the United States is one of the countries that produces the most publications in general. In addition, many sports management magazines are from the United States, which favors the proliferation of works on these topics from that country.

It is curious that the second country with the most articles is Spain, since this country does not have the base and structure of resources that the United States has. This indicates the great interest in sport among Spanish researchers, as well as the specialization of the country’s experts in these variables.

Daniel C. Funk could be considered as one of the greatest experts in sports management since he has published dozens of articles on this subject. According to the results of this study, he is also the author with the most published works on loyalty in the field of sport. Furthermore, he is the author with the most citations, which certifies the quality of his work. All the authors that appear in the list of results are major experts on the subject, since having more than seven publications on a specific topic is a sign of their knowledge on the subject. It is worth highlighting two authors: Heath McDonald, who appears in the top five for both number of publications and number of citations; and the Spaniard Jerónimo García-Fernández (eight articles), who appears in the top five for number of publications. He is likely to be one of the main reasons for Spain ranking second worldwide in number of publications.

Another conclusion derived from the results presented in this literature review is about the type of journals that publish on these topics. Unsurprisingly, focusing on specific sports management sources is the best option, since they are the ones that publish the most about loyalty in sport. However, other journals that may approach this topic and that may include call for papers on sports management should not be disregarded, even though sports management is not their main focus.

### Conclusion

The subject selected for this systematic analysis of the literature has a wide variety of works. Sport generates great interest in society and therefore in the scientific community as well. This work attempts to order and classify the theory on loyalty and sports management and identify the main shortcomings of the scientific literature on this topic.

### Limitations and Future Research

The loyalty antecedents analyzed in this work have a strong presence in the theory; however, there are very few articles that present all the variables. From 328 articles found, only five consider satisfaction, service quality, commitment, and trust. Therefore, the approach of models that explain loyalty in the sports field offer an excellent starting point for continued research into this variable.

## Data Availability Statement

The raw data supporting the conclusions of this article will be made available by the authors, without undue reservation.

## Author Contributions

All authors contributed to conception and design of the research, reviewed the theorical background, organized the database, performed the bibliometric analysis, wrote all the sections of the manuscript, contributed to manuscript revision, read, and approved the submitted version.

## Conflict of Interest

The authors declare that the research was conducted in the absence of any commercial or financial relationships that could be construed as a potential conflict of interest.

## Publisher’s Note

All claims expressed in this article are solely those of the authors and do not necessarily represent those of their affiliated organizations, or those of the publisher, the editors and the reviewers. Any product that may be evaluated in this article, or claim that may be made by its manufacturer, is not guaranteed or endorsed by the publisher.
